# Evaluating the usability of a cancer registry system using Cognitive Walkthrough, and assessing user agreement with its problems

**DOI:** 10.1186/s12911-023-02120-8

**Published:** 2023-01-30

**Authors:** Fatemeh Bagheri, Faezeh Abbasi, Mojtaba Sadeghi, Reza Khajouei

**Affiliations:** 1 Department of Health Information Sciences, Faculty of Management and Medical Information Sciences, Kerman University of Medical Sciences, Kerman, Iran; 2 Department of Health Information Technology, Faculty of Paramedicine, AJA University of Medical Sciences, Tehran, Iran

**Keywords:** Usability, Cognitive walkthrough, Registry system, User experience

## Abstract

**Objective/aim:**

Good design of cancer registry systems makes them easy to use, while poor design of their user interfaces leads to user dissatisfaction and resistance. The objective of this study was to evaluate the usability of a cancer registry system using Cognitive Walkthrough (CW) and to assess users' agreement with its usability problems.

**Methods:**

CW was used to evaluate the registry system. We developed a checklist to help evaluators speed up the evaluation process, a problems form to collect the usability issues identified by the evaluators, and a problems severity form to determine the severity of problems by the evaluators. The problems were classified into two categories according to the CW questions and the system tasks. The agreement of the users with the system problems was examined by an online questionnaire. Users' agreement with the problems was then analyzed using the Interclass Correlation Coefficient in the SPSS 22 (Statistical Package for Social Science).

**Results:**

In this study, 114 problems were identified. In the categorization of problems based on the CW questions, 41% (n = 47) of the problems concerned the issue of “users do not know what to do at each stage of working with the system”, 24% (n = 27) were classified as “users cannot link what they intend to do with system controls”, and 22% (n = 25) were related to “user's lack of understanding of the system processes”. Based on user tasks, about 36% (n = 41) of the problems were related to “removing patient duplication” and 33% (n = 38) were related to “registration of patient identification information”. User agreement with the problems was high (CI 95% = 0.9 (0.96, 0.98)).

**Conclusion:**

System problems often originate from user ignorance about what to do at each stage of using the system. Also, half of the system problems concern a mismatch between what users want to do and the system controls, or a lack of understanding about what the system does at different stages. Therefore, to avoid user confusion, designers should use clues and guides on the screen for users, design controls consistent with the user model of thinking, and provide appropriate feedback after each user action to help users understand what the system is doing. The high agreement of users with the problems showed that in the absence of users system designers can use CW to identify the problems that users face in the real environment.

**Supplementary Information:**

The online version contains supplementary material available at 10.1186/s12911-023-02120-8.

## Introduction

According to the World Health Organization, cancer is the second leading cause of death after cardiovascular disease worldwide [1]. The first step in controlling cancer is gathering information about the incidence, type, and location of the disease [2]. In this regard, the cancer registration system was developed. This system helps to better manage and control the disease by providing accurate information about its prevalence and patient survival [3].

One of the factors contributing to the success of an information system is its good design [4]. Good design is consistent with the goals, needs, and interests of users [5]. Given the difference in computer experience and expertise of users and the unfamiliarity of information systems developers with the user population, the design of these systems is not often tailored to the needs and interests of their users. Also, according to previous studies [6–9], poor design of information systems leads to users' fatigue, and dissatisfaction, as well as, system inefficiency and rejection. The good design of an information system contributes to its usability. According to ISO 9241-11, usability means that a system, product, or service can be used by specified users to achieve specified goals with effectiveness, efficiency, and satisfaction in a specified context of use [10]. Usability refers to aesthetic appearance, ease of use, easy navigation, user-friendliness, and clarity of the design of an information system [11]. Despite the increasing focus on usability and the development of relevant methods and procedures, information systems designers fail to design systems based on usability principles [9].

Usability evaluation methods fall into two user-based and expert-based categories. In the user-based evaluation methods, users participate in the evaluations to identify problems with information systems. Since recruiting a sufficient number of real users for user-based usability evaluation methods is sometimes difficult or expensive, it is essential to use other methods that identify usability problems hindering real users [12, 13]. In expert-based evaluation methods, experts identify usability problems in view of users' abilities [14]. Cognitive Walkthrough (CW) is one of the most common expert-based evaluation methods [15]. The main purpose of CW is to identify the simplest way to perform a task by creating an action sequence tree for the task and its sub-tasks. In this method, evaluators identify most usability problems of an interface and their causes especially severe problems easily, quickly, and at a low cost. Also, this method is suitable for evaluating information systems that their users are beginners [16]. However, it is unclear how much information system users agree with the problems identified by evaluators in such expert-based methods. Thus, examining the agreement of users with usability problems helps to improve the accuracy of the results of the expert-based evaluation methods, especially the CW method. So far, the agreement of information system users with the problems identified in expert-based usability evaluations has not been sufficiently studied. Among previous studies, only one study [17] measured the extent to which users agreed with the problems identified by the evaluators in a Heuristic Evaluation of an emergency information system. Other similar studies reported the results of CW evaluations [18–20], developed CW extensions such as Heuristic Walkthrough [21], compared CW with other expert-based methods such as Heuristic Evaluation [6, 22], compared CW with user-based methods [23, 24], or compared other expert-based evaluation methods, including Heuristic Evaluation with user-based methods [25, 26]. One of the information systems in the healthcare domain is the cancer registry system, which has been developed to improve the implementation and management of cancer registration programs. Good design and high usability of this system result in better effectiveness, efficiency, and user satisfaction of the system for users [10]. Therefore, the objective of this study was to evaluate the usability of a cancer registry system using CW and to assess users' agreement with the problems identified by CW.

In this study, first, the usability problems of a cancer registry system were identified using CW. Next, the severity of the problems was determined by the evaluators using a severity rating scale. Then, the problems with higher severities were given to users for determining the severities by this group. Finally, users' agreement with the problems identified by CW was assessed.

## Research methodology

### System and setting

The cancer registry system is a national information system that was implemented in 2015 and is currently used by more than one hundred users in twenty universities in Iran. The cancer registry system allows the collection and management of information on cancer patients and the calculation of the incidence and prevalence of cancer in different geographical areas. Registration of patients' profiles and their tumor information, authentication, removing patient duplications, and annual reporting of patients' information are among the capabilities of this system. The most frequent and routine tasks of the system are “Recording patient's personal details and tumor information” and “Removing patient duplications”. The present study was performed in the cancer registration center of Kerman University of Medical Sciences (KUMS).

This study was conducted in two phases:

#### Phase 1. Evaluation of the cancer registry system using CW

**Fig. 1:**
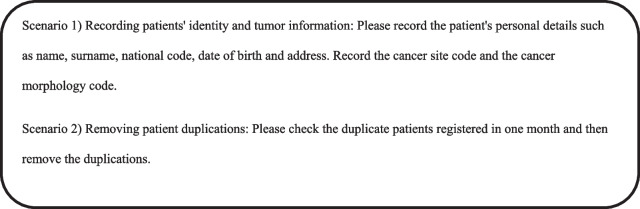
Two routine scenarios in cancer registration

**Fig. 2:**
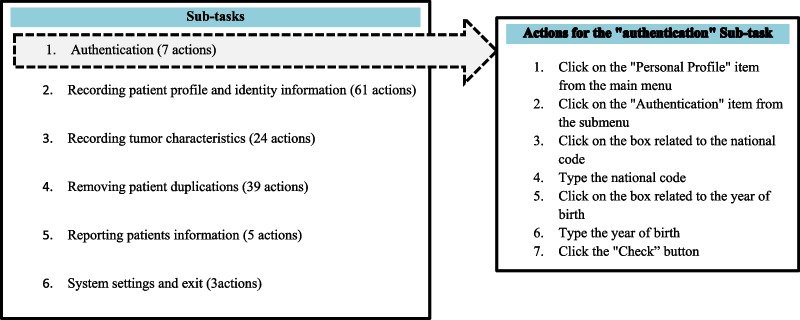
List of sub-tasks related to the two scenarios and an example of actions requires for completing the “Authentication” sub-task

**Fig. 3:**
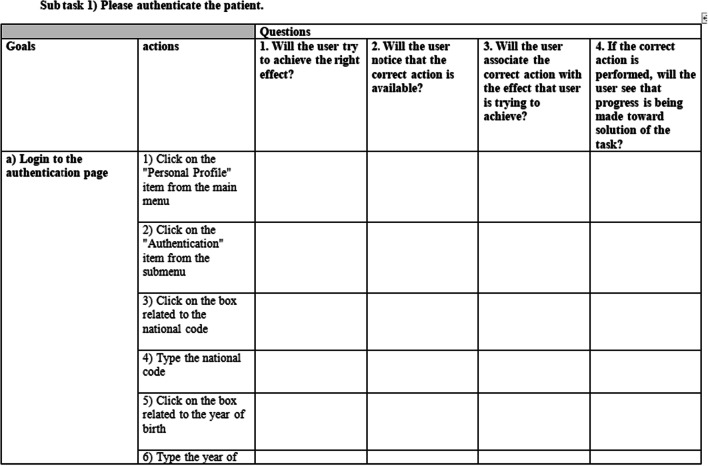
A part of the checklist developed to help evaluators speed up the evaluation process and to reduce the likelihood of error

The CW method introduced by Polson and Lewis [27, 28] was used for the evaluation. This phase consisted of three steps: 1. evaluation preparation, 2. evaluation execution and 3. analyzing the problems. In the preparation step, with the assistance of an expert familiar with the workflow of the cancer registry system, two scenarios were developed based on two frequent and routine tasks of the system users (Fig. 1). Performing these scenarios in the system required the completion of six sub-tasks. The sub-tasks and the actions required to complete each sub-task in the system are listed in Fig. 2. Finally, to speed up the evaluation process and to reduce the likelihood of error, a checklist was developed based on the CW method for evaluating the execution of the sub-tasks and their actions in the system. Then, to confirm the validity of prepared scenarios and defined sub-tasks, this checklist was reviewed by an expert familiar with the cancer registry system and a medical informatics expert. This checklist included the sub-tasks, the user's purpose for completing the sub-tasks, the actions needed to complete the sub-tasks, and the four questions recommended to be asked for the evaluation of each action in CW (1. Will the user try to achieve the right effect?, 2. Will the user notice that the correct action is available?, 3. Will the user associate the correct action with the effect that the user is trying to achieve?, and 4. If the correct action is performed, will the user see that progress is being made toward the solution of the task?) [20]. Overall, this checklist included 6 sub-tasks, 139 actions, and 556 questions (139 * 4) (Fig. 3) (see Additional file 1).

**Fig. 4:**
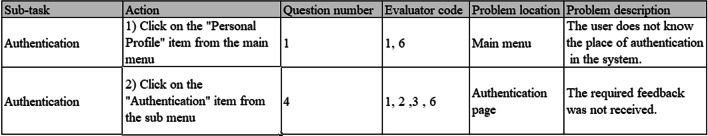
A part of the problems form used for collecting problems identified by the evaluators

In the second step, since a sample size of 10 ± 2 is sufficient to detect 80% of the usability problems of a system [30–33], eight evaluators were invited to perform the evaluation. These evaluators had a background in medical informatics and health information technology and received at least six months of training and experience in using CW. Before the evaluation, evaluators held a joint meeting with an evaluation expert having more than ten years of experience in evaluating health information systems. In this meeting, the procedure of evaluation was discussed and all evaluators worked with the cancer registry system and got acquainted with the routine process of executing scenarios in the system. To begin the evaluation, paper-based copies of the checklist were distributed among the evaluators. The evaluators independently performed the sub-tasks listed in the checklist, took the actions one by one, and determined how easily users can work with the system, taking the system's design and feedback, as well as, the users’ background and knowledge into account. The answer to the checklist questions was “yes” if no problem was detected, and “no” if there was a problem in the system design. Whenever the answer to any of the questions was no, the evaluators entered a description of the problem into the checklist. In the third step, a problems form was designed to collect the problems identified by the evaluators. In this form, each problem was described with six features including sub-task, action, question number, evaluator code, error location, and error description (Fig. 4) (see Additional file 2). To analyze the problems, the questions with yes answers by all evaluators were discarded, but questions to which at least one of the evaluators gave a no answer were included in the problems form for further consideration. Finally, by combining duplicate items in the problems form, the final problem list was formed.

**Fig. 5:**
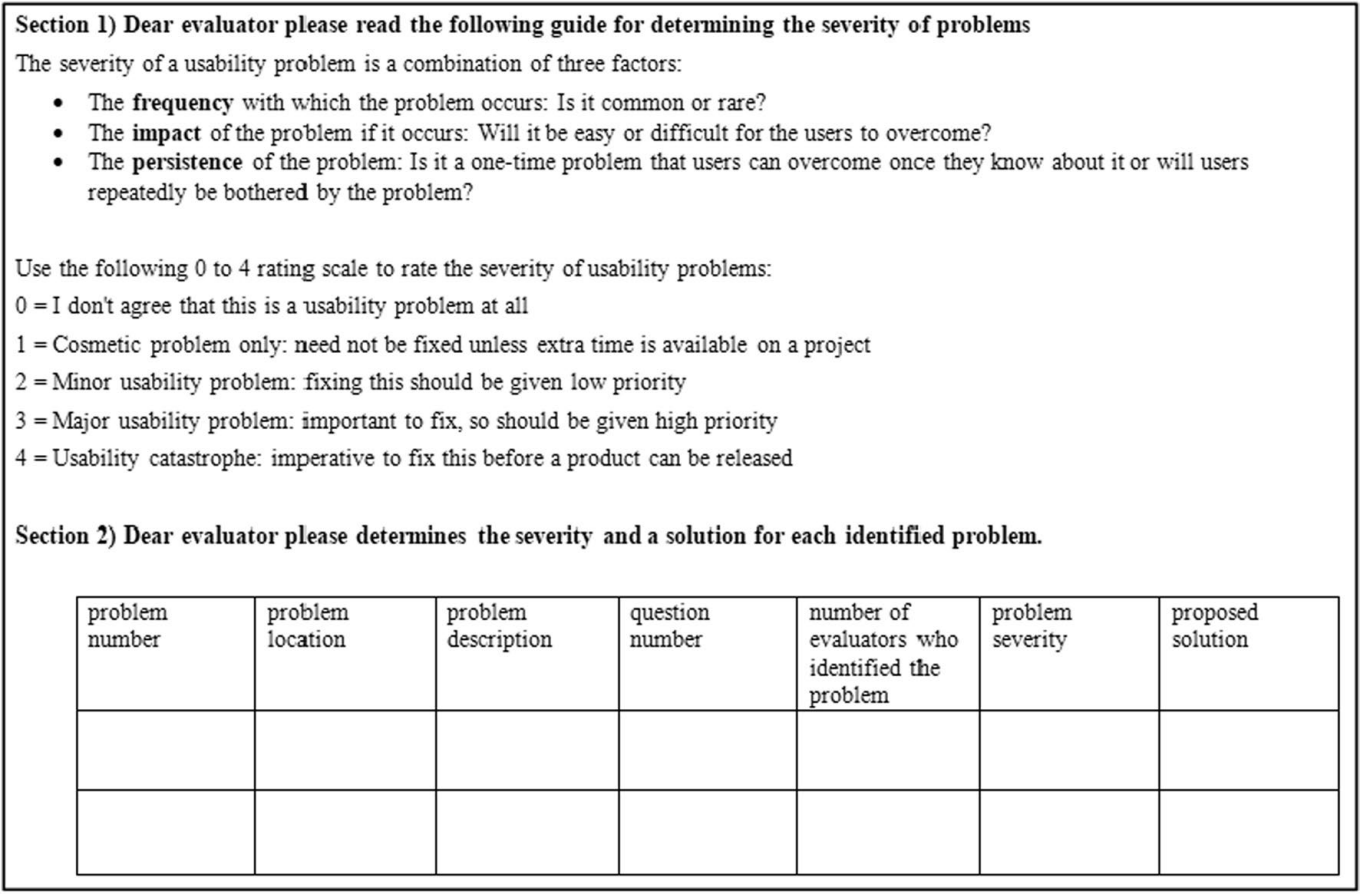
A part of the problems severity form

To determine the severity of usability problems of information systems, among several severity rating method [34–36], we used a common severity rating method proposed by Nielsen that prioritizes the problems in terms of their effect on user interaction [31, 36–39]. In this method, the severity of problems is determined based on three criteria of frequency, impact, and persistence [40]. Thus, to determine the severity of problems by the evaluators, a problems severity form was developed by the researchers based on Nielsen severity rating. This form consisted of two parts. The first part was a guide for determining the severity of the problems according to Nielsen severity rating. The second part included the problem number, problem location, problem description, question number, number of evaluators who identified the problem, problem severity, and solution. Using this form, evaluators independently assigned a score from 0 to 4 to each problem (0 = not a problem. 1 = cosmetic problem, does not need to be fixed. 2 = minor problem, low priority for fixing 3 = major problem, high priority for fixing. 4 = usability catastrophe, imperative to be fixed) (Fig. 5) (see Additional file 3). To determine the final severity of each problem, the mean severity of that problem was calculated. In the end, the problems were categorized based on evaluation questions. For example for the question, “Will the user try to achieve the right effect?” the category was created under the heading “Notifying of action at hand”. Therefore, the problems were divided into four categories: “Notifying of action at hand”, “Existence of necessary controls”, “Visibility and clarity of controls for users” and “User perception of the work process in the system”. To be able to prioritize the tasks based on their difficulty and their importance for fixing, the problems were also categorized by tasks. Upon this categorization, the problems were divided into five categories: “Authentication”, “Recording patient identity information”, “Recording tumor characteristics”, “Removing patient duplications” and “Reporting patient information”. To investigate how the complexity of a sub-task can hinder the successful completion of that sub-task by users, the relationship between the number of actions in each sub-task and the number of problems users encountered when performing the sub-task was tested using Spearman's test.

#### Phase 2. Examining user agreement with the problems identified by the evaluators in the cancer registry system

**Fig. 6:**
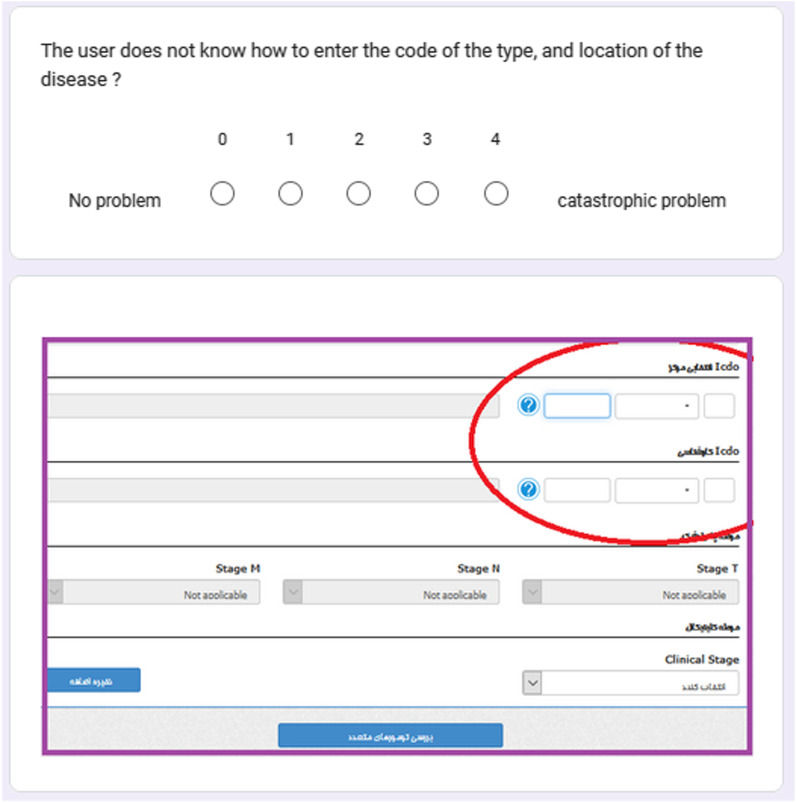
A sample of the questions used to assess the agreement rate of users with problems identified by CW

**Fig. 7:**
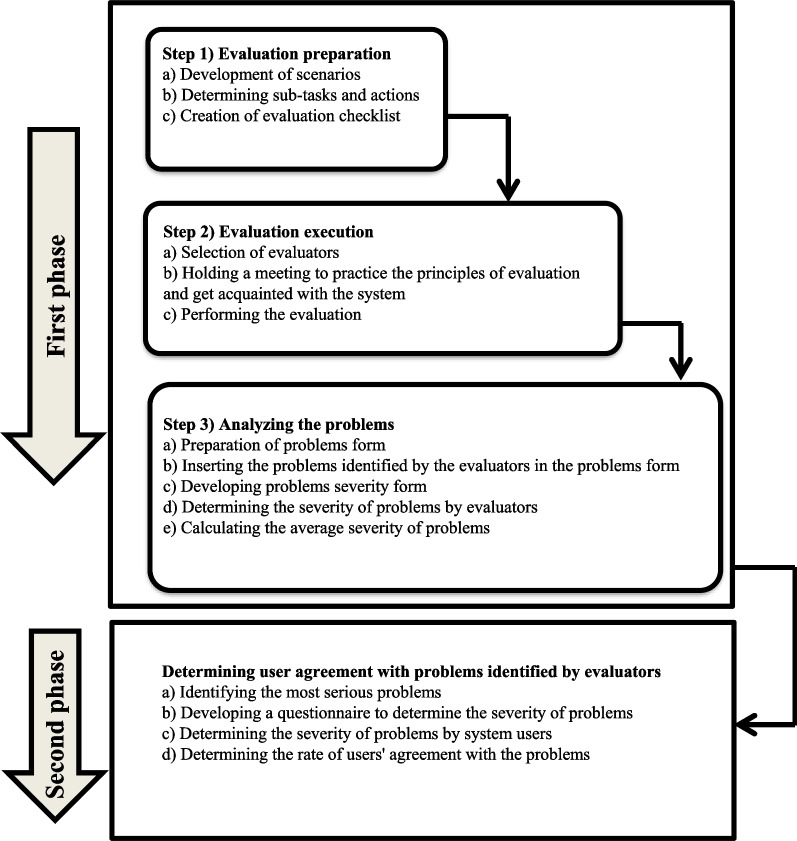
The study workflow of CW evaluation and assessing user agreement with the identified problems

An online questionnaire was developed (in Google Forms) to assess the agreement of users with problems identified in this study. Due to the large number of problems, and to increase the response rate of users the severity cut-off was set at 3.5 so that only problems with higher severities (severity > 3.5) were included in the questionnaire (n = 32). This questionnaire had two sections. The first section included ten questions concerning age, gender, education, computer-related degree, ability to work with a computer, experience of using personal computers, the rate of internet use, work experience in cancer registration, and the experience of working with cancer registry system and other health information systems. Except for the age, the answers to the rest of the questions were multiple choice. The second section contained 32 problems with high severity. These problems were multiple choice with zero (no problem) to four (catastroph problem, imperative to fix) options. To better present and clarify the location of each problem, a screenshot of the system where the problem occurred was placed after each problem. The content and face validity of the questionnaire were confirmed by three medical informatics specialists. The reliability of the questionnaire was confirmed by Cronbach's alpha of 93% (Fig. 6) (see Additional file 4). The purpose of usability studies is to identify the problems that users encounter during interaction with an infomation system. CW is a task-based method that focuses on the usability of system for users who would work directly with the system. Since, IT experts contribute to the development and maintenance of information systems and health professionals are not familiar with most of the tasks of information systems, this study has only focused on the real users of the system. Thus, the participants in this stage were users of the cancer registry system who work in the cancer registration centers. The following two criteria were used to select the study participants, (1) having more than a month of work with the cancer registry system, and (2) voluntary participation. To collect the data, the participating users were provided with the questionnaire link via WhatsApp or SMS (Short Message Service). The purpose of the study was mentioned in the introduction of the questionnaire, and the participants were ensured that the confidentiality of their information would be maintained. To obtain informed consents, the participants were asked to answer the questionnaire only if they were willing to participate in the study (Fig. 7).

To analyze the data for this step, the data were extracted from Google Forms in Excel format and imported into SPSS 22 (Statistical Package for Social Science). The Interclass Correlation Coefficient was then used to calculate the coefficient of participant agreement using the mean scores that participants gave to each question and the mean severities of these issues. The study workflow is shown in Fig. 7.

## Results

### Phase 1. The CW evaluation of the cancer registry system

The evaluators’ demographic information is shown in Table 1. Most of the evaluators (75%) were women, less than 30 years old, and had a master's degree. Also, more than half (62%) of the evaluators were familiar with health information systems and had the experience with information systems evaluation.

**Table 1:** Evaluators’ demographic information

Demographic information	n (%)
*Age*	
< 30	6 (75)
>= 30	2 (25)
*Sex*	
Male	2 (25)
Female	6 (75)
*Education degree*	
Master’s	6 (75)
PhD	2 (25)
*Acquaintance with health information systems*	
Medium	3 (37.50)
High	5 (62.50)
*Experience of information systems evaluation*	
Medium	3 (37.50)
High	5 (62.50)

Evaluators identified 435 problems during CW of the registry system. After merging problems and removing duplicates, 114 problems remained (see Additional file 5).

#### Categorizing problems based on evaluation questions

The categorization of problems based on evaluation questions along with the number and mean severity of problems are shown in Table 2. About 90% of the problems were minor or mojor. Most of the problems were related to the “notifying of action at hand” category. The categories “Visibility and clarity of controls for users” and “User perception of the work process in the system” each accounted for approximately 25% of the problems. The mean severity of problems in these categories was almost at the same level, between 1.9 and 2.2. The lowest number of problems and also the lowest mean severity of problems were related to the “existence of necessary controls” category.

**Table 2:** Categorization of problems based on evaluation questions and mean problem severity

Category based on evaluation questions	n (%)	Severity n (%)				Average severity
		Cosmetic	Minor	Major	Catastrophe	
Notifying of action at hand	47 (41.22)	2 (4.25)	22 (46.80)	16 (34.04)	7 (14.89)	2.16 ± 0.80
Existence of necessary controls	15 (13.15)	0	11 (73.33)	4 (26.66)	0	1.92 ± 0.50
Visibility and clarity of controls for users	27 (23.68)	0	10 (37.03)	16 (59.25)	1 (3.70)	2.21 ± 0.53
User perception of the work process in the system	25 (21.92)	1 (4)	10 (40)	11 (44)	3 (12)	2.17 ± 0.74
Total		3 (2.63)	53 (46.49)	47 (41.22)	11 (9.64)	

#### Categorizing problems based on the scenarios

The categorization of the problems by sub-tasks, along with the number and mean severity of problems, is shown in Table 3. The mean severity of the problems in each sub-task was at almost the same level, between 1.8 and 2.6. The highest number of problems were related to the “Removing patient duplications” and “Recording patient identity information” sub-tasks. Using the Spearman test, no correlation was observed between the number of actions and the number of problems per task (p > 0.05, Spearman correlation = 0.75).

**Table 3:** Categorizing usability problems by tasks

Sub-task	n (%)	Severity n (%)				Average severity
		Cosmetic	Minor	Major	Catastrophe	
Authentication	1 (9.64)	0	0	1 (100)	0	2.56 ± 0.09
Recording patient identity information	38 (33.33)	3 (7.89)	23 (60.52)	9 (23.68)	3 (7.89)	1.87 ± 0.71
Recording patient tumor specification	29 (25.43)	0	11 (37.93)	16 (55.17)	2 (6.89)	2.28 ± 0.67
Removing patient duplications	41 (35.96)	0	18 (43.90)	17 (41.46)	6 (14.63)	2.27 ± 0.66
Reporting patients information	2 (1.75)	0	0	2 (100)	0	2.69 ± 0.26
System settings and exit	3 (2.63)	0	2 (66.66)	1 (33.33)	0	0.61 ± 2.21
Total		3 (2.63)	54 (47.36)	46 (40.35)	11 (9.64)	

### Phase 2. Examining the agreement of users with the problems identified by CW

Out of 101 cancer registry users, 27 (26.73%) users responded to the questionnaire. The demographic information of these twenty-seven participants is shown in Table 4. In general, about 70.4% (n = 19) of the participants were female and a third of the participants were younger than 30 and a third were between 40 and 49 years old. More than half of the participants (51.8%) had a bachelor's degree, computer-related education (55.6%), and an advanced level of computer skills (62.9%). About 37% of the participants (n = 10) stated that they use a personal computer daily and about 85% (n = 23) stated that they use the Internet for two to several hours a day. Forty-four percent of the participants (n = 12) had between 2 and 10 years of experience in the cancer registration unit of a Medical Sciences University. More than half of the participants (n = 14) stated that they have one to two years of experience using the cancer registry system. Most of the participants (n = 24) also had a history of using other health information systems.

**Table 4:** Demographic information of the participants

Demographic information	Number (%)
*Age*	
< 30	9 (33.33)
30–39	6 (22.22)
40–49	9 (33.33)
>= 50	3 (11.11)
*Gender*	
Male	8 (29.62)
Female	19 (70.37)
*Education degree*	
Bachelor's	14 (51.85)
Master’s and higher	13 (48.14)
*Computer-related education degree*	
Yes	15 (55.55)
No	12 (44.44)
*Computer skills*	
Elementary or intermediate	10 (37.03)
Advanced	17 (62.96)
*Computer use*	
Daily	10 (37)
Two to several times a week	9 (33.3)
Two to several times a year	4 (14.9)
Having no access to computer, except at working place	4 (14.8)
*Internet use*	
Two to several hours a day	23 (85.2)
Less than one hour a day	4 (14.8)
*Work experience in cancer registry*	
Less than a year	3 (11.1)
Between 1 and 2 years	9 (33.3)
Between 2 and 10 years	12 (44.4)
More than 10 years	3 (11.1)
*Experience with the cancer registry system*	
Less than 6 months	4 (14.8)
Between 6 months to 1 year	2 (7.4)
Between 1 and 2 years	7 (25.9)
More than 2 years	14 (51.9)
*History of using other health information systems*	
Yes	24 (88.9)
No	3 (11.1)

**Fig. 8:**
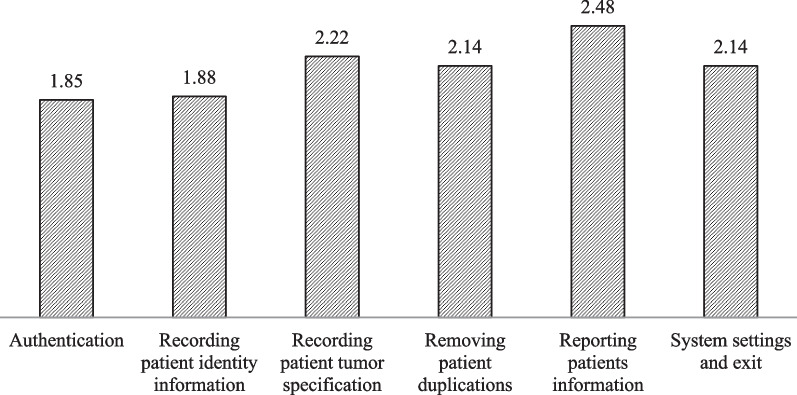
The severity of usability problems from the users' point of view

The users' agreement with the usability problems identified by CW was CI 95% = 0.9 (0.96, 0.98). The most severe problems were related to the “Reporting patients information” (2.48) in the system. Problems concerning “Authentication” of a patient (1.85) and “Recording patient identity information” including manual entry of patient's date of birth, displaying inappropriate format for patient's date of birth after manual entry, the impossibility of manual entry of characteristics such as nationality, province of birth, and improper system alert for changing the language (1.88) had the lowest severity (Fig. 8).

## Discussion

### Principal findings

In this study, evaluators identified 114 usability problems related to the design of a cancer registry system. From the evaluators' point of view, in categorizing problems based on CW questions, most of the problems were related to the fact that a user can not set a goal at each stage of working with the system. Mismatch of the system controls with the action at hand, and unclear feedback about what the system is doing, each accounted for about a quarter of the problems. In the categorization of problems based on user tasks, about three-quarters of the problems concerned the tasks of “removing patient duplications” and “recording patient identity information”. Twenty-seven users participated in the second phase of the study in order to check their agreement with the usability problems. These users strongly agreed with the problems identified by CW.

### Evaluation of the cancer registry system using CW

In this study, eight evaluators identified a large number of system usability problems in a CW evaluation. This result is in line with the results of other studies indicating that CW can identify a large number of usability problems in an information system with 3–8 evaluators [6, 20, 41].

In categorizing problems based on CW questions, most of the usability problems emerged since a user could not set a goal at each stage of working with the system. This result is in line with the results of a study [20] that used CW to evaluate a maternity information system and showed that less than a quarter of the problems are related to the lack of knowledge concerning the action at hand at every stage of working with the system. One of the reasons for such problems is the lack of instructions on using the system or the lack of clues to guide the user through the system, which makes it difficult to find a way to work with the system, imposes a cognitive burden on their memory, and leads to fatigue and dissatisfaction of users with information systems [42].

In this study, a quarter of the problems arose due to the inconsistency of system controls such as icons, buttons, and menus with their intended actions. This result confirms the results of a previous study evaluating a remote drug delivery system by heuristic and CW evaluations, which showed that it is difficult for a user to find the relationship between system controls and their corresponding actions. For example, unclear categorization of the user interface elements confuses users on how to navigate the available options. According to other studies [43–46], problems such as small size and closeness of icons and buttons, inappropriate labels, small and inconsistent fonts, irrelevant colors, vague and obscure menus, and Improper placement of controls are among design problems that prevent users from understanding the relationship between system controls and corresponding actions. Ideally, the design of the controls in a system's interface should match the users' mental model to facilitate users’ interpretation of the controls' functions. Therefore, system designers can minimize users’ memory load by using meaningful and understandable symbols on the interface [47, 48].

According to studies [20, 22, 49], CW can identify problems related to the users’ understanding of system performance. In this regard, almost a quarter of the problems identified in the present study showed that the users do not have a proper understanding of what the system does at different stages. A previous study [49] revealed that poor system response, vague feedback or lack of feedback, and even delays in receiving feedback result in a difficult understanding of system performance. Therefore, providing appropriate audio and video feedback after each user’s action prevents confusion and user errors, and ultimately improves performance.

Categorization of problems according to tasks showed that most problems were associated with the tasks having a higher number of actions compared to other tasks. The highest number of problems in the cancer registry system evaluated in this study occurred during “recording patient identity” such as name, surname, age, and address of residence; as well as during the process of “removing patient duplications”. This result confirms the results of two former studies that evaluated a drug information system [9], and a health research system [50]. These studies have shown that tasks that require more action raise more problems for users. Hence, these tasks increase the cognitive load of the user's memory and require more time to be completed. Thus, it is recommended to reduce the number of actions required to perform tasks in the development and redesign of the system.

### Agreement of the cancer registry users with the problems identified by CW

In expert-based methods, experts primarily identify general problems related to the user interface. But a user-testing method only identifies problems that users encounter when performing their tasks [51]. Also, expert-based methods identify a higher number of problems compared to user-testing methods [51, 52]. The agreement of the cancer registry users with the problems identified by CW was used to investigate to what extent users confirm the issues identified by CW. According to the results, users have a high agreement with the problems identified by CW. In previous studies, the users' agreement with the problems identified by CW has not been measured. The only paper [17] that addressed the agreement of users with the results of such studies has shown that users strongly agree with the problems identified by Heuristic Evaluation. Other relevant studies measured the agreement between the results of the CW method and the results of the other user-centered evaluation methods. These studies that evaluated self-care mobile applications for patients with diabetes [24] and Acquired Immune Deficiency Syndrome (AIDS) [23] have shown that CW and think—aloud identify similar problems and users and evaluators strongly agree on the required user interface modifications such as changes in color, font, and text size. Besides, our study confirms the results of studies that measured the agreement between the results of other expert-centered evaluation methods and the results of user-centered evaluation methods [25, 26]. These studies that evaluated a virtual reality program on eating behavior [26] and the self-care system of AIDS patients [25] have reported a high agreement between the results of the Heuristic Evaluation and user-centered evaluation methods.

### Study implications

The results of this study and previous studies have shown that the lack of a help function for using the system or the lack of clues to guide the user confuses users about what to do at each stage of working with the system. Therefore, to avoid user confusion, system designers and developers embed the help functionality in a suitable place in the system and use clues to facilitate navigation throughout the system. When the user could not associate the intended actions to their controls in the system, this usability problem imposes a heavy cognitive load on the user's memory. On the other hand, poor system response and lack of appropriate and timely feedback prohibit understanding the performance of the system. Hence, system developers should design meaningful and understandable controls based on users' mental models and design and provide appropriate audio and video feedback for each user action, to help the user understand what the system is doing.

According to the results of this study, most problems originate from tasks that require a higher number of actions. Tasks with several actions in an information system entail long-term interaction with the system or moving between different pages, which impose a heavier cognitive burden on the user's working memory [53]. Therefore, it is recommended to develop systems that allow users to perform their tasks by taking fewer actions.

One of the challenges of task-based evaluation methods such as CW is the inappropriate selection of tasks in the preparation phase of the evaluation. Inappropriate selection of tasks may prevent the identification of important and frequently encountered usability problems [28]. Usability problems, especially the most frequent ones, increase users' cognitive load and lead to fatigue and burnout [54]. Therefore, identifying the recurring problems helps improve the design of information systems and increases system performance by reducing the cognitive burden and reducing the learning time for users. In this study, we evaluated a cancer registry system by selecting the most routine and frequent tasks performed by users. Therefore, the problems identified in this study are the most important problems of the system from the users' point of view in the real environment.

Due to the large number of problems identified by CW in this study, we measured the degree of users' agreement solely with more severe problems. Our results indicate a high degree of user agreement with the identified problems. These results are in line with the results of a study [17] that evaluated an emergency information system and showed that the level of user agreement with the problems identified by Heuristic Evaluation is high. Therefore, whenever access to real users is difficult, expert-based evaluation methods such as CW can be used effectively to evaluate information systems and find usability problems.

### Study limitations and future studies

This study had four limitations. First, The main objective of this study was to assess the agreement rate of users with usability problems identified by the CW method. Therefore, some of the usability problems that users may encounter in a real environment could be overlooked. Future studies can identify all the system usability problems faced by users through user testing methods. Second, participating users were provided only with the usability problems identified by CW. Subsequent studies can use open-ended questions to determine whether users add other problems not identified by CW. Third, to increase the accuracy of answers and response rate, from the large number of problems identified in the CW evaluation, only the problems with higher severities were provided to users. Studies with a lower number of problems, can measure user agreement with all problems. Fourth, we invited all the users of the cancer registry system in Iran to participate in this study. Despite sending several reminders to users to participate in the study, the response rate was not high. Examining the agreement of a higher number of users with usability problems can increase the accuracy of the results. However, this is the first study examining the agreement of users with the results of a CW of a healthcare information system and the results can be used for evaluating similar systems.

## Conclusion

In categorizing problems based on CW questions, most of the system problems are related to the issue that the user cannot set a goal at each stage of working with the system. Therefore, system designers and developers should provide a help function in a convenient location of the interface and use informational clues to facilitate the navigation of users throughout the system. In addition, half of the problems indicated that the user cannot make an accurate connection between his goal and the system controls, or he does not have a proper understanding of what the system is doing at different stages. Therefore, to prevent user confusion, controls should be used that match user's mental model, and the system should provide appropriate feedback after each user action to help the user understand what the system is doing. Since most problems occur when completing tasks with a higher number of actions, minimizing the number of actions in each task improves the design and usability. In this study, the scenarios were developed based on the most routine and frequent tasks of users. Therefore, the results can be used for designing systems having similar tasks. Due to the high agreement of users with the problems identified by CW, the results of this study indicate that in the absence of users, the CW can detect a large number of problems, especially important problems that may be encountered by users in the real environment.

## Supplementary Information

**Additional file 1**. Evaluation checklist.

**Additional file 2**. Problems form.

**Additional file 3**. Problems severity form.

**Additional file 4**. Online questionnaire.

**Additional file 5**. Identified problems.

## Data Availability

The data generated and analyzed during this study are available from the corresponding author upon reasonable request.

## Abbreviations

CW: Cognitive Walkthrough
SPSS: Statistical Package for Social Science
SMS: Short Message Service
AIDS: Acquired Immune Deficiency Syndrome
KUMS: Kerman University of Medical Sciences
