# Supplementary material for: Evaluating the usability of a cancer registry system using Cognitive Walkthrough, and assessing user agreement with its problems
Source: BMC Med Inform Decis Mak. 2023 Jan 30;23:23. doi: 10.1186/s12911-023-02120-8 (PMC9887869; doi:10.1186/s12911-023-02120-8)
Supplement: Supplementary file 1 — Additional file 1. Evaluation checklist. [file 12911_2023_2120_MOESM1_ESM.pdf]

Title: Evaluation checklist

Sub task1) registering the profile and identity information of a new cancer patient:

Dear evaluator, please use the pathology report provided to you to record the patient's profile using the list below and answers the four questions mentioned for each of the actions.

		Questions			
**Goals**	**actions**	**1. Will the user try to achieve the right effect?**	**2. Will the user notice that the correct action is available?**	**3. Will the user associate the correct action with the effect that user is trying to achieve?**	**4. If the correct action is performed, will the user see that progress is being made toward solution of the task?**
**a) Login to the system**	1) Open the browser				
	2) Click on the URL box				
	3)Enter the system address in the browser to log in				
	4) Click on the box in front of the ID				
	5) Enter ID				
	6) Click on the box next to the password				
	7) Enter the password				
	8) Click on the login button				

**b) Searching for a patient with a national code**	9) Click on the box in front of the national code				
	10)Enter the patient's national code				
	11) Click on the search button				
**c) Searching for a patient by name, surname and gender**	Please delete the national code and this time search with the patient's first and last name.				
	12) Click on the box in front of the name				
	13) Enter the name				
	14) Click on the box in front of the last name				
	15) Enter the last name				
	16) Click on the gender box				
	17) Placing the mouse on gender from the opened list				
	18) Click on gender				
	19) Click on the search button				
	If you did not find the desired patient				

	through the search, register the patient's profile as a new patient by following the steps below.				
**d) Registration of personal information**	20) Click on the individual profile item in the left menu				
	21) Placing the mouse on the patient profile registration item				
	22) Click on the patient profile registration item				
	23) Click on the box in front of the national code				
	24) Enter the national code				
	25) Ensuring the correctness of the national code				
	26) Click on the box in front of the name				
	27) Enter the name				
	Please enter last name and gender independently from the checklist.				

	28) Click on the box in front of the date of birth				
	29) Click on the left and right buttons above the date table that appears				
	30) Click the mouse on the desired day				
	*Please delete the date entered in the date box and enter the date manually this time using the following steps.				
	31) Click on the box in front of the issue date				
	32) Hand typing the date in the box				
	33) Click on the nationality box				
	34) Manual entry of nationality: Enter the nationality manually.				
	*Delete the nationality and enter it this time with the following				

	action.				
	35) Click on the desired nationality from the opened list				
	36) Click on the box in front of the province of birth				
	37) manually type the province of birth in this box				
	38) Scrolling in the opened list				
	39) Placing the mouse on the desired province				
	40) Click on the province				
	* Choosing the city of birth and occupation is the same as the previous item, so you fill these two items independently from the checklist.				
**e) Registration of residence details**	41) Placing the mouse on the page scroll				
	42) double click on the scroll and drag it down				

	43) Click on the box in front of the province				
	44) hand typing the province				
	45) Place the mouse on the scroll in the opened list				
	46) Double click on the scroll and drag it down				
	47) Placing the mouse on the desired province				
	48) Click on this province				
	*Due to the similarity of the items of the city and the area of residence with the item of the province, these two in this				
	Checklists are not included. Therefore, please fill these items yourself independently of the checklist.				
	49) Placing the mouse on the				

	address box				
	50) Click on this box				
	51) Type address				
	* Leave these items blank due to the similarity between the address and the phone, mobile phone and postal code.				
	52) Click on "Add to address list" button				
	53) Click on the "Close" button in the message that appears				
	* There is no need to fill in the insurance details.				
	54) Click twice on the scroll corner of the page				
	55) Dragging the scroll to the bottom of the page				
**f) Verification of patient information**	56) Click on the button "Confirm patient information"				
	*If the starred items are left				

	empty, you will receive a message. Thus, upon seeing this message, perform actions 57 to 60, and otherwise go to action 61.				
	57) Click on the "close" button in the message				
	58) Double click on the scroll and drag it upwards				
	59) Filling in the remaining items (Fill in these items without the checklist.)				
	60) Click again on the button "Confirm patient information"				
	61) Click on the button to confirm the "successful registration" message.				

Sub task2) registering the characteristics of the patient's tumor: Now it is time to register the patient's cancerous tumor. Please register the tumor report on the same page of the system using the following checklist

		Questions			
**Goals**	**actions**	**1. Will the user try to achieve the right effect?**	**2. Will the user notice that the correct action is available?**	**3. Will the user associate the correct action with the effect that user is trying to achieve?**	**4. If the correct action is performed, will the user see that progress is being made toward solution of the task?**
**a) Login to the cancer tumor registration page**	1) Click on the button "Register patient reports"				
**b) Examining the patient's personal information and ensuring their accuracy**	2) Checking the patient's profile in the box				
	As you can see, the patient's address field is empty, so you must fill this field with the following actions				
	3) Click on the box below "Patient's address at the time of occurrence"				
	4) Click on one of the addresses that appear based on the address of the pathology report				
**c) recording tumor characteristics**	5) Scroll to the bottom of the page and be in the registered reports box				
	6) Click on the				

	"diagnosis method" box.				
	7) Click on the desired diagnostic method				
	In the rest of this section, just fill in the box related to the update date, the name of the center and the file number. be completed independently of the checklist due to repetitiveness.				
	In the "Pathology/Clinical Report" box, the pathology report should be scanned and copied into this box, which we will skip due to the limited facilities.				
	8) Click on the first box from the left under the section "ICDO selection center"				
	9) manual typing of cancer site code (C				

	code)				
	*Please delete the entered code and this time find the code by applying the following.				
	10) Scroll in the opened list				
	11) Click on the desired M code in the opened list				
	12) Click on the second box				
	13) manual typing of morphology code (M code)				
	14) Click on the third box				
	15) Type the desired grade code				
	Please fill in the box related to "Bachelor's ICDO" independently of the checklist and with the same recent code.				
	Please select the item "Uncertain" in "Clinical Stage" independently from the checklist.				
	16) Click on the				

	button "Additional storage"				
	17) Click on the "Close" button in the message that appears				
	18) Click on the "check multiple tumors" button				
	19) Click on the "Close" button in the message				
	20) Scroll to the bottom of the page				
	21) In the "Final Tumors List" table, select the report you have just registered with a mouse click.				
	22) Click on the button "Final storage of the tumor"				
	23) Click "Close" in the message that appears				
	24) Click on the "Final registration" button.				

Sub task3) repetition: In this part, repetition should be done both for the individual and for the individual's cancerous tumors. In this way, repeat the operation using the following actions. (Repeat for the same patient whose report you registered.

		**Questions**			
**Goals**	**actions**	**1. Will the user try to achieve the right effect?**	**2. Will the user notice that the correct action is available?**	**3. Will the user associate the correct action with the effect that user is trying to achieve?**	**4. If the correct action is performed, will the user see that progress is being made toward solution of the task?**
**a) Searching for a specific patient among the patients to repeat the list of patients**	1) Hover over "Cancer Profile" in the left menu				
	2) Click on the item "Cancer specifications"				
	3) Click on the sub-item "Annual list of patients"				
	4) Click on the box under "Select Year"				
	5) Manually type the desired year				
	6) Click on the desired year from the opened list				
	7) Click on the search box below				
	8) Type name and surname				
	9) Click on the display button				
	10) Click on the				

	desired item under the menu				
	11) Click on the display button				
	12) Place the mouse on the scroll in the corner of the page				
	13) Double click on it				
	14) Scroll down				
	15) Ensuring that no repetition is performed by checking the repetition column, the number of tumors and the final registration for the desired patient.				
**b) Placing the desired patient in the repeat queue**	16) Click on the icon "go to the repeat queue" (blue icon)				
	17) Click on the gender box				
	18) Click on the desired gender				
	19) Click on the search button				
	20) Placing the mouse on the				

	scroll				
	21) Double click on scroll				
	22) Scroll down				
**c) Removing the patient from the repetition queue**	23) Click on the icon "Exit from the repetition queue" (orange icon)				
	24) Click on the "Yes" button				
	25) Click on the "Close" button.				
**c) Go to the profile page of the person and registered reports**	26) Click on the "Cancer registration" button. (green button)				
	27) Double click on the scroll and drag it down				
	28) Select the report you registered by clicking on the "registered reports" table.				
	29) Scroll to the bottom of the page				
	30) Click on the button "Examination of multiple tumors"				
	31) Click on				

	"Close" button				
	32)Scroll to the bottom of the page				
	33) In the table, click on the last recorded report in terms of date.				
	34) Click on the bottom box "Patient's address at the time of occurrence"				
	35) Click on the desired address under the opened menu				
	36) Click on the button "Final storage of the tumor"				
	37) Click on "Close" button				
	38) Click on the "final registration" button				
	39) Click on "Close" button				

Sub task4) receiving the list of patients: Please find the list of patients registered in 2014 and May.

		**Questions**			
**Goals**	**actions**	**1. Will the user**	**2. Will the user**	**3. Will the user**	**4. If the correct**

		try to achieve the right effect?	notice that the correct action is available?	associate the correct action with the effect that user is trying to achieve?	action is performed, will the user see that progress is being made toward solution of the task?
**a) Entering the registered patients search page**	1) Click on the item "Cancer details" from the left menu				
	2) Click on "Registered patients form"				
	3) Click on the box under "year"				
	4) Click on the desired year (2014)				
	Due to the similarity, complete the rest of the checklist items independently				
	5) Click on the "Show" button.				

Sub-task 5) please authenticate the patient.

		Questions			
**Goals**	**actions**	**1. Will the user try to achieve the right effect?**	**2. Will the user notice that the correct action is available?**	**3. Will the user associate the correct action with the effect that user is trying to achieve?**	**4. If the correct action is performed, will the user see that progress is being made toward solution of the task?**
**a) Login to the authentication page**	1) Click on the "Personal Profile" item from the main menu				
	2) Click on the "Authentication" item from the sub menu				
	3) Click on the box related to the national code				
	4) Type the national code				
	5) Click on the box related to the year of birth				

	6) Type the year of birth				
	7) Click the "Check" button				

6) Make the necessary settings from the settings menu and exit from the system

		**Questions**			
**Goals**	**actions**	**1. Will the user try to achieve the right effect?**	**2. Will the user notice that the correct action is available?**	**3. Will the user associate the correct action with the effect that user is trying to achieve?**	**4. If the correct action is performed, will the user see that progress is being made toward solution of the task?**
**a) Entering the settings page**	1Click on the "Settings menu" item from the left menu				
**a) Find the exit button and exit the system**	2-Click on the "Settings menu" item from the left menu				
	3-Click on "Exit				
