# Supplementary material for: Evaluating the usability of a cancer registry system using Cognitive Walkthrough, and assessing user agreement with its problems
Source: BMC Med Inform Decis Mak. 2023 Jan 30;23:23. doi: 10.1186/s12911-023-02120-8 (PMC9887869; doi:10.1186/s12911-023-02120-8)
Supplement: Supplementary file 2 — Additional file 2. Problems form. [file 12911_2023_2120_MOESM2_ESM.pdf]

Title: problems form

Problems identified by evaluators:

Sub-task	Action	Question number	Evaluator code	Problem location	Problem description
