# Supplementary material for: Evaluating the usability of a cancer registry system using Cognitive Walkthrough, and assessing user agreement with its problems
Source: BMC Med Inform Decis Mak. 2023 Jan 30;23:23. doi: 10.1186/s12911-023-02120-8 (PMC9887869; doi:10.1186/s12911-023-02120-8)
Supplement: Supplementary file 3 — Additional file 3. Problems severity form. [file 12911_2023_2120_MOESM3_ESM.pdf]

Title: problems severity form

Section 1) Dear evaluator please read the following guide for determining the severity of problems

The severity of a usability problem is a combination of three factors:

- The **frequency** with which the problem occurs: Is it common or rare?
- The **impact** of the problem if it occurs: Will it be easy or difficult for the users to overcome?
- The **persistence** of the problem: Is it a one-time problem that users can overcome once they know about it or will users repeatedly be bothered by the problem?

Use the following 0 to 4 rating scale to rate the severity of usability problems:

0 = I don't agree that this is a usability problem at all

1 = Cosmetic problem only: need not be fixed unless extra time is available on a project

2 = Minor usability problem: fixing this should be given low priority

3 = Major usability problem: important to fix, so should be given high priority

4 = Usability catastrophe: imperative to fix this before a product can be released

Section 2) Dear evaluator please determines the severity and a solution for each identified problem.

problem number	problem location	problem description	question number	number of evaluators who identified the problem	problem severity	proposed solution
