# Supplementary material for: Evaluating the usability of a cancer registry system using Cognitive Walkthrough, and assessing user agreement with its problems
Source: BMC Med Inform Decis Mak. 2023 Jan 30;23:23. doi: 10.1186/s12911-023-02120-8 (PMC9887869; doi:10.1186/s12911-023-02120-8)
Supplement: Supplementary file 4 — Additional file 4. Online questionnaire. [file 12911_2023_2120_MOESM4_ESM.pdf]

Title: online questionnaire

An online questionnaire was developed (in Google Forms) to assess the agreement of users with problems identified in this study. You can access the questionnaire with the link below:

https://docs.google.com/forms/d/e/1FAIpQLScDsiN1PFv7113KuLuFUQrPS2pTBaZ9Zo8pLy2N0kg-dHglZQ/viewform?usp=sf_link
