# Supplementary material for: Evaluating the usability of a cancer registry system using Cognitive Walkthrough, and assessing user agreement with its problems
Source: BMC Med Inform Decis Mak. 2023 Jan 30;23:23. doi: 10.1186/s12911-023-02120-8 (PMC9887869; doi:10.1186/s12911-023-02120-8)
Supplement: Supplementary file 5 — Additional file 5. Identified problems. [file 12911_2023_2120_MOESM5_ESM.pdf]

Title: identified problems

Number	Problem
**1**	Lack of proper feedback when entering the wrong user code
**2**	Lack of proper feedback when entering the wrong password
**3**	To type the patient's first name in Persian, the system gives an error message In the search section
**4**	To type the patient's last name in Persian, the system gives an error message In the search section
**5**	The search item is not clear on the search page and the user does not know what to search based on
**6**	Lack of proper feedback from the system when entering the national code
**7**	Lack of proper feedback for wrong or without results
**8**	The user does not know what to do to register the patient's personal information.
**9**	The user does not know how to be sure of the correctness of the national code
**10**	There is no control to check national code by the user
**11**	The correctness mark of the national code is ambiguous for the user.
**12**	To type the patient's first name in Persian, the system gives an error in the registration section of personal information
**13**	The user does not know how to enter the date of birth
**14**	The control required to enter the date of birth is missing.
**15**	The user cannot make a connection between the action to be taken and the system controls to record individual information.
**16**	The user does not know how to select the date in the date table
**17**	The user does not receive proper feedback after selecting the day.
**18**	The user does not know that he can type the date in the desired date selection box
**19**	To insert the date, the necessary controls and format are not suitable
**20**	The user cannot understand the necessary connection between the empty box and what he should type in it.
**21**	After wrongly entering the date in the system, the user does not receive proper feedback.
**22**	When the nationality drop-down menu is opened, the user does not know that he can type it.
**23**	Lack of necessary controls for typing nationality
**24**	The unintelligible connection between the box and typing nationality
**25**	Despite the possibility of typing "nationality", nothing is displayed in the search box and the system does not provide feedback to the user.
**26**	To type "province of birth", the user does not know that he can type it. Because it is displayed as a drop-down list.
**27**	There is no appropriate place to type "place of birth"
**28**	Typing the province of birth and the opened list are unrelated, and the user receives an incomprehensible English message.
**29**	Failure to see "City" after typing it by the user and failure to receive appropriate feedback from the system to the user for this solve problem
**30**	Not seeing the scroll on the side of the screen by the user and not understanding the appropriate action to see the continuation of the information
**31**	The user does not know that it is no longer possible to type the province from the system drop-down menu to select "province".

**32**	There are no controls for typing "province".
**33**	The user cannot make a connection between the open list and the possibility of typing
**34**	After typing "province", the desired province is not placed in the relevant box and the system does not give the user proper feedback.
**35**	The user does not know what to do to register the "address".
**36**	The connection between the button and what the button does is unclear to the user
**37**	After registering the address, an unclear message is displayed and the system does not provide proper feedback.
**38**	Due to misplaced buttons, the user often forgets to press the button to continue.
**39**	The design of the "register patient reports" button is inappropriate and due to its large size, it looks more like a box and the user does not know what to do.
**40**	With a label that has a button, the connection between the user's goal and what the button does is not clear
**41**	By pressing the button and entering a new page, the user does not know what page she/he has entered
**42**	Lack of necessary control to check the national code registered by the user
**43**	After viewing the "Personal Profile" page, the user may continue without checking the information again. There is no feedback on what the user should do here.
**44**	Lack of proper warning and feedback to complete the information on the tumor report page
**45**	To register the code, there are three boxes in front of the user, and the user does not know which box to start from to enter the codes. None of the boxes have labels. There is no explanation or guidance.
**46**	The type format is unclear to the user.
**47**	The user does not know that in order to register the disease code, he must complete other fields in addition to the first field<br>The user assumes that the system automatically enters the second field.
**48**	The user does not know that all M codes are in the list and can find it from the list. The shape of the box does not look like a drop-down list at all. It is not necessary to enter M next to the code, but because the user does not know, he always enters this M, so more time is taken from him.
**49**	There was no necessary control to select the code. First, the user must enter at least one character of the code so that the list of codes appears for him.
**50**	The connection between the M code field and what the user wants to do is not clear. The user is confused.
**51**	After selecting the M code, the feedback was not received correctly.
**52**	The code typing format is unclear for the user and the user does not know how to type the code.
**53**	After typing the code, if the code is not selected by the mouse, the desired code does not appear in the box and the system does not have proper control over this.
**54**	The user does not understand the use of the third box to select the code and does not know what to enter here.
**55**	The connection between the code that the user must enter and the type of box is not clear to the user.
**56**	The code input format is not clear for the user. No guide or label for the box
**57**	The save button has an inappropriate design and the user does not know what to do to save the information.
**58**	The button "Check for multiple tumors" is not clear on the screen, and the user does not know whether it is necessary to press this button at any stage of working with the system.
**59**	Ambiguity in the names of the buttons makes the user not know what the function of the

	button is.
**60**	When the user presses the check multiple tumors button, the system does not give proper feedback.
**61**	The user doesn't know that he/she should use the "final tumor list" table in the system to get the relevant report.
**62**	There is no necessary control to select the report in the table.
**63**	When selecting a report, the user cannot make a connection between the control and the work she is doing.
**64**	By selecting the report in the table, inappropriate feedback is presented in the system.
**65**	Ignoring the "final save tumor" button by the user because the labels are not clear.
**66**	Due to the busy design, the button "final save tumor" may not be seen by the user.
**67**	What the "final save tumor" button does is unclear.
**68**	After pressing the "final save tumor" button, the user does not know exactly what happened and what to do.
**69**	The user does not know what to select from the "Cancer Characteristics" sub-section.
**70**	The relationship between reduplicating and the item "cancer profile" is unclear to the user.
**71**	When clicking on the "Annual List of Patients" button, the user does not know what to do here
**72**	The connection between repetition and entering the annual list page is unclear for the user.
**73**	There is no control for typing "year".
**74**	The user does not know that before filling in the box below the search, he must click the display button. The order of filling the items is not clear. Also, the button is not in a good place
**75**	When clicking the "Show" button, what the button does is not understandable to the user.
**76**	After pressing the button on the listing page, no clear feedback is received.
**77**	The user does not know what to enter in the search box and does not know at all that he/she should fill this box after clicking the show button and seeing the annual list..
**78**	In the listing page, the relationship between the tags and what the user wants to do is not clear.
**79**	The system does not provide any feedback when typing the name and surname manually.
**80**	The user does not know how to repeat after finding the name of the patient.
**81**	The relationship between repetition and the numbers zero and one on the page is unclear.
**82**	The "go to a repeat queue" icon is small and without a label, and the user does not know what this icon does.
**83**	By clicking on the "go to repeat queue" icon, the user will not notice what is happening.
**84**	On the profile page that opens, all fields are filled except for the gender field. The user may miss this topic and not fill it.
**85**	The user does not know the need to search for the patient twice.
**86**	By pressing the search button, the user does not get feedback because the information appears at the bottom of the page and the user does not realize this until he goes to the bottom of the page.
**87**	The user does not know what into do after bringing the patient in the repeated queue?
**88**	The connection between what the button does and the user's intent is not understood. Why should the patient leave the repeat queue after entering the repeat queue?
**89**	After pressing the button, the user does not know what exactly happened. It is difficult for the user to understand the process.
**90**	By clicking on the "Cancer registration" icon, the user does not know what to do.
**91**	By clicking the "Cancer registration" icon, the connection between the function of the button and the user's goal is not clear.
**92**	By pressing the button, the user enters a page, but does not understand the process and what happened.

**93**	The user does not know that in the table of registered reports he must select the report in the table. While there is no guide or explanation at the top of the table.
**94**	By selecting the report, the user will not notice the system feedback and will only see the selected report.
**95**	When clicking the multi-tumor scan buttons, the user does not know whether to scan multiple tumors or not.
**96**	The user cannot see the "Check multiple tumors" button in the indexing step.
**97**	Due to the unclear label of the check multiple tumors button on the list page, the user does not understand its function.
**98**	The user cannot clearly see the last report recorded in the table to select it.
**99**	The user does not know what the concept of "address at the time of update" means.
**100**	In the process of repeating, the user does not know why he should press the "final save" button when he has already saved all of this.
**101**	The relationship between what it does and what it aims to do is not between concepts.
**102**	The reason for the final registration for the user is not understood in the act of repetition
**103**	By clicking on the "Cancer Details" item from the initial menu, the directory path is unclear to the user.
**104**	By clicking "Registered patients form" from the initial menu, the directory path is unclear for the user.
**105**	The user does not know where to go for authentication?
**106**	By pressing the review button, the required feedback is not received and a warning message is always displayed.
**107**	There is no control necessary to continue the configuration process.
**108**	The user does not know that to exit, the user must follow the path of the setting.
**109**	The user cannot understand the connection between the logout button and the settings button and does not know that he has to press the settings button to log out.
**110**	By clicking on the left and right buttons above the date table, the button above the table cannot be seen and the user does not know what to do.
**111**	By clicking on the left and right buttons above the date table, there is no necessary button to insert the date
**112**	By clicking the left and right buttons above the date table, the user does not understand the connection between the date she must enter and the table.
**113**	By clicking the left and right buttons above the date table, the system does not give feedback after entering the date.
**114**	The scroll is not clear when you double click on the scroll and drag it down.
